# Contemporary Theory and Research

**Published:** 1893-08-12

**Authors:** 


### Contemporary Theory and Research.

### RE-VACCINATION.

Among the many rather drastic provisions of the new French Sanitary Code, which has excited considerable opposition, not the least remarkable is that which makes re-vaccination compulsory in the course of the eleventh and twenty-first year. Parents and guardians are held personally responsible for the carrying out of this measure, and may be congratulated on this new addition to their duties.

### THE POPULATION OF PARIS.

M. Lagneau has presented a report to the Académie de Médecine proving by very elaborate statistics the following facts: That owing to the enormous mortality of newly-born Parisians put out to nurse, owing to the great mortality of infants, principally among the labouring classes, from athrepsy, diptheria, and measles, owing to the high mortality of the citizens from phthisis and other tuberculous affections, the average life or average age of those who die in Paris appears to be only 28 years, while for the whole of France the average age is 40. Nevertheless, the population of Paris increases more and more by the incessant immigration of provincials and foreigners who make up two-thirds, as against one-third of natives. But owing to their feeble natality and high mortality, the families which are not united to these immigrants die out in three, four, or at most five generations.

### INFANT MORTALITY IN BIRMINGHAM.

According to the report of Dr. Hill on the health of the city of Birmingham for 1892, it seems that though the health of the city is, on the whole, satisfactory, and the mortality below the average of large towns, the infants are badly off. The chief causes of death among them are found to be in the digestive organs, and the etiology of these may be found in deprivation of mothers' milk, due to the employment of women away from their homes, or maternal neglect, and in the use of improper and injurious substitutes. Dr. Hill considers the remedy is to be sought in social rather than sanitary agencies, and, like most persons who have studied this question, strongly urges the regulation of infant life insurance. The view that married women should not be permitted by law to be employed in factories is upheld, and it seems that this rule is enforced in his works by one employer of labour on a very large scale near Birmingham. It would doubtless have a salutary effect on the home if other employers followed suit, but general legislation being hardly possible, the remedy must be sought, as Dr. Hill points out in the slow process of better teaching and example.

### THE CONTAGION OF LOCK JAW.

M. Verneuil in his researches into the communication of tetanus shows that the bacillus of Nicolaïer is found very often in the earth of the stables on high roads. It is rarely found, on the contrary, among the sand of the shore. At Biarritz and at Cannes tetanus is unknown; but the oyster fishers of Arcachon, whose naked feet are often wounded by the broken oyster shells, often get tetanus on their way back over the infected high road. In the same way the negroes of Havannah often take tetanus from the little wounds which the "jigger" makes in the heel. Certain ponds are well known to give tetanus to any horse whose skin is abraised, and a case is cited of a newly-born infant bound with linen dipped in pond water who succumbed to tetanus. They examined the water of the pond and found it teeming with these bacilli. A horse may even be tetaniferous without having tetanus. A horseman contracted the disease by biting his own tongue. It was found that the hairs of the horse he rode were inoculated, and that the dust disengaged as he rode had infected the tiny wound. Sheep and oxen are also liable in the same way to communicate the disease without having it themselves. The remedy is to be sought in the rigid disinfection of all suspected wounds without enlarging them.

### PENTAL AS AN ANESTHETIC.

Wood and Cern draw attention to two singular effects of pental: First, analgesia is produced before consciousness is lost, the patient executes all the doctor's orders, feels the touch of the instrument but experiences no pain. All the same, it would be imprudent to operate before the complete loss of consciousness. Most of the accidents which have occurred after the employment of pental are due to violent reaction taking place in the patient who has not completely succumbed to the influence of the drug. Second, ten to fifteen minutes after the first administration of pental it can be employed again without its action being enfeebled; it seems even as if the patient were more easily affected. The patient must lie down during the operation or dangerous accidents may occur. In the majority of cases the patient recovers consciousness quietly, and experiences no ill effects whatever. In small operations two minutes are required for the narcose, two for the operation, and two for the restoration of the patient to his normal state. Most of the pental inhaled is eliminated without any modification by the lungs.

